# Prospective Associations of Lifetime Post-traumatic Stress Disorder and Birth-Related Traumatization With Maternal and Infant Outcomes

**DOI:** 10.3389/fpsyt.2022.842410

**Published:** 2022-07-22

**Authors:** Julia Martini, Eva Asselmann, Kerstin Weidner, Susanne Knappe, Jenny Rosendahl, Susan Garthus-Niegel

**Affiliations:** ^1^Department of Psychiatry and Psychotherapy, Faculty of Medicine of the Technische Universität Dresden, Dresden, Germany; ^2^Institute of Clinical Psychology and Psychotherapy, Technische Universität Dresden, Dresden, Germany; ^3^Differential and Personality Psychology, Faculty of Health, HMU Health and Medical University Potsdam, Potsdam, Germany; ^4^Department of Psychology, Faculty of Life Sciences, Humboldt-Universität zu Berlin, Berlin, Germany; ^5^Department of Psychotherapy and Psychosomatic Medicine, Faculty of Medicine, Technische Universität Dresden, Dresden, Germany; ^6^Evangelische Hochschule Dresden, University of Applied Sciences for Social Work, Education and Nursing, Dresden, Germany; ^7^Institute of Psychosocial Medicine, Psychotherapy and Psychooncology, Jena University Hospital, Jena, Germany; ^8^Institute for Systems Medicine (ISM) and Faculty of Human Medicine, MSH Medical School Hamburg, Hamburg, Germany; ^9^Institute and Policlinic of Occupational and Social Medicine, Faculty of Medicine of the Technische Universität Dresden, Dresden, Germany; ^10^Department of Child Health and Development, Norwegian Institute of Public Health, Oslo, Norway

**Keywords:** lifetime PTSD, birth-related traumatization, pregnancy, postpartum, infant outcomes

## Abstract

**Objective:**

Many women experience traumatic events already prior to or during pregnancy, and delivery of a child may also be perceived as a traumatic event, especially in women with prior post-traumatic stress disorder (PTSD). Birth-related PTSD might be unique in several ways, and it seems important to distinguish between lifetime PTSD and birth-related traumatization in order to examine specific consequences for mother and child. This *post-hoc* analysis aims to prospectively examine the relation of both, lifetime PTSD (with/without interpersonal trauma) and birth-related traumatization (with/without postpartum depression) with specific maternal and infant outcomes.

**Methods:**

In the prospective-longitudinal Maternal in Relation to Infants' Development (MARI) study, *N* = 306 women were repeatedly assessed across the peripartum period. Maternal lifetime PTSD and birth-related traumatization were assessed with the Composite International Diagnostic Interview for women. Maternal health during the peripartum period (incl. birth experience, breastfeeding, anxiety, and depression) and infant outcomes (e.g., gestational age, birth weight, neuropsychological development, and regulatory disorders) were assessed *via* standardized diagnostic interviews, questionnaires, medical records, and standardized observations.

**Results:**

A history of lifetime PTSD prior to or during pregnancy was reported by 25 women who indicated a less favorable psycho-social situation (lower educational level, less social support, a higher rate of nicotine consumption during pregnancy). Lifetime PTSD was associated with pregnancy-related anxieties, traumatic birth experience, and anxiety and depressive disorders after delivery (and in case of interpersonal trauma additionally associated with infant feeding disorder). Compared to the reference group, women with birth-related traumatization (*N* = 35) indicated numerous adverse maternal and infant outcomes (e.g., child-related fears, sexual problems, impaired bonding). Birth-related traumatization and postpartum depression was additionally associated with infant feeding and sleeping problems.

**Conclusion:**

Findings suggest that both lifetime PTSD and birth-related traumatization are important for maternal and infant health outcomes across the peripartum period. Larger prospective studies are warranted.

**Implications:**

Women with lifetime PTSD and/or birth related traumatization should be closely monitored and supported. They may benefit from early targeted interventions to prevent traumatic birth experience, an escalation of psychopathology during the peripartum period, and adverse infant outcomes, which in turn may prevent transgenerational transmission of trauma in the long term.

## Introduction

Many women experience traumatic events already prior to or during pregnancy, and delivery of a child may also be perceived as a traumatic event, especially in women with prior post-traumatic stress disorder (PTSD). PTSD is one of the most debilitating mental disorders and the 12-month prevalence in the German general population is 2.3% (95%CI: 1.8–2.9) (1). Women are more often affected by PTSD than men (12 month-rate in women and men: 3.6%; 95%CI: 2.8–4.7; and 0.9%, 95%CI: 0.6–1.5) and most of them experience traumatic events already prior to pregnancy (1). Still, the peripartum period is considered to be a vulnerable time frame for the onset, recurrence, and exacerbation of PTSD symptoms and other mental disorders in women (e.g., peripartum anxiety and depressive disorders) (2). Due to traumatic experiences prior to or during pregnancy about 3.3% of pregnant women suffer from PTSD and prevalence rates in risk populations (e.g., history of intimate partner violence) are even higher (about 18%) (3). In addition, up to one-third of women who have recently given birth describe their birth experience as traumatic (4), up to 10% suffer from clinically relevant posttraumatic stress symptoms during the first weeks thereafter (5–8), and up to 4% develop the full clinical picture of PTSD (3, 7, 9, 10). The prevalence of postpartum PTSD is even higher in at-risk populations (e.g., up to 19% after preterm delivery, emergency cesarean section, or still birth) (3, 7, 9, 10).

Birth-related PTSD might be unique in several ways, and it seems important to distinguish between lifetime PTSD and birth-related traumatization. Birth experience may become traumatic if the birth involves actual or threatened death or an injury for a woman or for the infant (2, 11, 12). Birth-related traumatization affects the mother-child-dyad and childcare of the baby might trigger maternal traumatic memories of birth. Given that childcare is usually associated with an increased vigilance, the hyperarousal criteria should be considered with caution. Risk factors of birth-related traumatization are a history of sexual trauma and intimate partner violence, depression or anxiety in pregnancy, fear of childbirth, complications during pregnancy, obstetric interventions/ operative delivery, peripartum infant complications, a subjective negative birth experience, and perception of inadequate intrapartum care or lack of social support (5, 11–14).

Both, lifetime PTSD and birth-related traumatization may be associated with postpartum depressive and anxiety disorders, whereas the relation to pregnancy- and child-related fears is less studied (5, 15–17). Moreover, PTSD in the context of pregnancy and childbirth might affect the partnership (e.g., sexual problems) and the mother-child-dyad (e.g., bonding) (16, 18, 19). For instance, traumatic memories or re-experiencing of the childbirth might reduce emotional availability of the mother to the infant, especially if the infant serves as a trigger of the event. This, in turn, could be crucial for the type of attachment the infant develops to the mother. Mother-child dyads may be further fragiled by the numbness affected women are likely to perceive (20). Some studies showed that birth-related PTSD symptoms are associated with bonding and breastfeeding difficulties that may have long-term health implications for infants (21, 22). In addition, symptoms of hyperarousal and intrusion might lead to impaired childcare and interaction with the child (e.g., angry or intrusive interaction) (19, 23, 24) and comorbid maternal depression might contribute additionally to parenting impairment (25, 26). A review by Christie et al. (27) found that parental PTSD was associated with impaired functioning across various parenting domains and less optimal parent-child relationships (27). The Perinatal Interactional Model of Intergenerational transmission of traumatization proposes that maternal PTSD leads to suboptimal caregiving behavior and parent-child interactions, which undermine child regulatory capacity and increase distress leading to poorer social-emotional outcomes for offspring of parents with PTSD (28). Moreover, Pat-Horenczyk et al. (29) suggested the idea of relational emotional regulation in which difficulties with emotion regulation pass from mother faced with trauma to the child (29). In line with this, Davies and colleagues showed that women with postpartum PTSD symptoms following childbirth and postpartum depression perceive their attachment relationships to be less optimal and rated their infants as being temperamentally more difficult and less easy to soothe as compared to non-symptomatic women (9). Moreover, some prospective studies found maternal postpartum PTSD to be associated with difficult temperament and/ or poorer cognitive and social-emotional development in infants up to 2 years (19, 30). Especially interpersonal trauma might be crucial for the relation between mother and child and for early child development (e.g., neuropsychological development) (2, 31, 32).

### Aims of the Study

In sum there is evidence that PTSD prior to and during pregnancy as well as birth-related traumatization are associated with adverse maternal and infant outcomes. As it has been argued that birth-related PTSD might be unique in several ways it seems important to distinguish between lifetime PTSD and birth-related traumatization in order to examine specific consequences for the mother and child (31, 33, 34). A *post-hoc* analysis of the data of the *Ma*ternal *A*nxiety in *R*elation to *I*nfant Development (MARI) study was conducted to examine specific associations of lifetime PTSD and birth-related traumatization with the above mentioned outcomes. In the prospective-longitudinal MARI study (35, 36) women were recruited already during early pregnancy and reported their lifetime diagnostic status prior to pregnancy. Participants were followed up three times during pregnancy and three times after delivery within this multi-wave and multi-method study. Given that anxiety and depressive disorders occur frequently in expectant mothers and may be also relevant for the considered outcomes, we chose expectant mothers with no anxiety and/or depressive disorders prior to or during pregnancy as reference group. This allows the examination of specific associations of lifetime PTSD and birth-related traumatization with the specific outcomes.

Using the data of the MARI study we were able to examine (1) whether women with PTSD prior to or during pregnancy were at higher risk for poor maternal health during the peripartum period (e.g., pregnancy-related fears, maternal anxiety and depressive disorder, operative delivery), for a traumatic birth experience, and adverse gestational and infant outcomes (e.g., regulatory disorders, attachment, neuropsychological development) as compared to women with neither anxiety nor depressive disorder until third trimester of pregnancy. Further, we examined (2) whether women suffering from birth-related traumatization were at higher risk for adverse maternal and infant outcomes (e.g., child-related fears, maternal anxiety and depressive disorder, maternal sexual problems, infant regulatory disorders) as compared to women with no anxiety or depressive disorder prior to delivery and no birth-related traumatization. Here we additionally investigated the role of postpartum depression at 2, 4 or 16 month postpartum (25).

## Methods

### Procedure

The prospective-longitudinal MARI Study was conducted among *N* = 306 expectant mothers, sampled from the community in gynecological outpatient settings in Dresden, Germany (study period: 01/2009 – 09/2012). Participating pregnant women (and their infants) completed up to seven assessments: T1 (baseline): week 10 to 12 of gestation; T2: week 22 to 24 of gestation; T3: week 35 to 37 of gestation; T4: 10 days postpartum; T5: 2 months postpartum; T6: 4 months postpartum; T7: 16 months postpartum (36).

Informed consent was obtained from all participants and all legal guardians of the infants. The MARI Study was carried out in accordance with the Helsinki Declaration (2013) and was reviewed by the Ethics Committee of the Technische Universität Dresden (No: EK 94042007). More detailed information including objectives, methods, design, and a detailed study flow chart has been published elsewhere (35, 36).

### Participants

Overall, *N* = 533 pregnant women were approached by the study team in gynecological outpatient settings in Dresden (Germany) and screened for inclusion and exclusion criteria. Fifty women were excluded based on the exclusion criteria, which were as follows: gestational age >12 weeks (*n* = 8), younger than 18 or older than 40 years (*n* = 8), multiple pregnancy (*n* = 2), history of more than three spontaneous abortions/(induced) terminations of pregnancy/stillbirths or infant impairment (*n* = 2), invasive fertility treatment (*n* = 9), severe physical disease/microsomnia/skeletal malformation (*n* = 6), substance abuse or heroin substitution during the past 6 months (*n* = 0), severe psychiatric illness (*n* = 2), expectation to leave the area of Dresden (*n* = 6), and insufficient mastery of German language (*n* = 7). Additional 9 women did not participate due to spontaneous abortion before the baseline interview (T1), 10 due to lacking consent of the father of the infant, 154 due to lack of time, and 4 due to unknown reasons (36).

Overall, data of 306 women were eligible for the MARI study. Due to spontaneous abortion/induced termination of pregnancy, the participation of *n* = 8 women ended after T1. During the study, *n* = 3 women moved away, *n* = 5 women could not be reached any more by phone, postal, or personal contact, *n* = 9 women reported lack of time or interest for further participation, and *n* = 7 women refused contact for follow-up assessment. Overall, retention rate until 16 months after delivery (T7) was 89.5% (*n* = 274). Some women did not participate at single assessments, e.g. due to preterm delivery, own/infant sickness, or lack of time (T2: *n* = 0, T3: *n* = 10, T4: *n* = 2, T5: *n* = 5, T6: *n* = 1, T7: *n* = 7) (a detailed flow chart of the MARI study was presented by Martini et al. (36).

### Measures and Procedures

**Figure 1:**
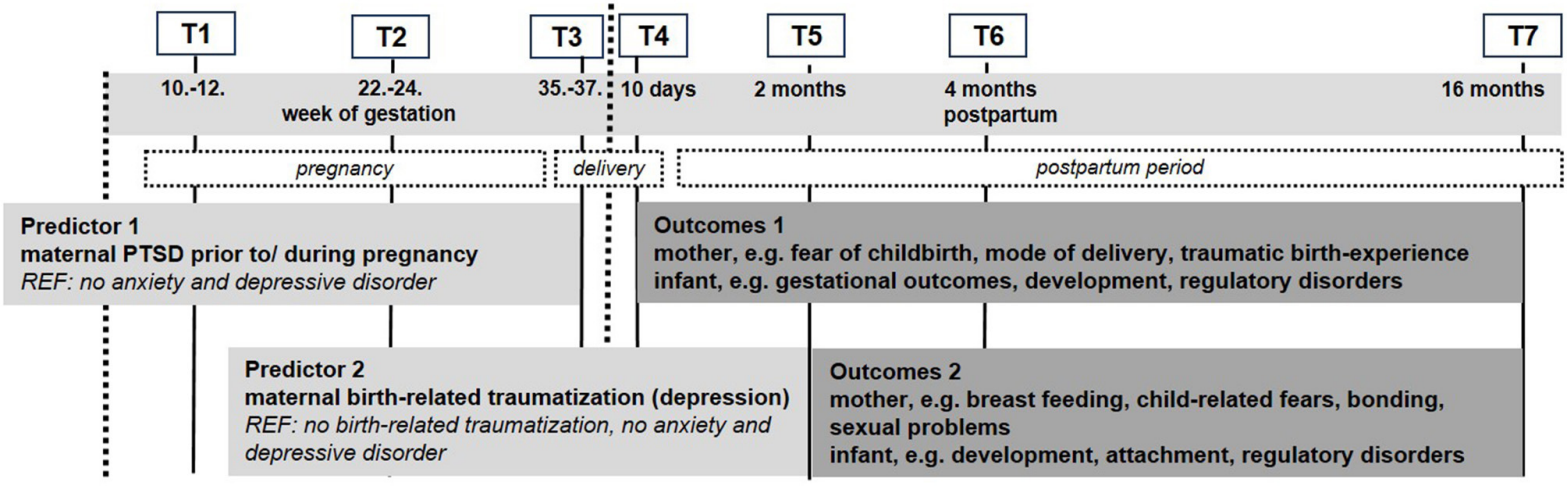
Assessment waves, measures and outcomes for the *post-hoc* analyses of lifetime PTSD and birth-related traumatization with maternal and infant outcomes.

Figure 1 presents the assessment points of the MARI study and predictors and outcomes of the present analyses. A Computer-Assisted Personal Interview (CAPI) version of the Composite International Diagnostic Interview for Women (CIDI-V) (37) was applied at each assessment wave except for T4 (10 days postpartum). Due to the special situation after delivery, questionnaires were used at T4 instead of the diagnostic interview to assess mode of delivery, birth experience, neonatal outcomes, and postpartum adjustment.

#### Predictors: PTSD Prior to or During Pregnancy and Birth-Related Traumatization

Maternal post-traumatic stress disorder as well as anxiety and depressive disorder were assessed using the CIDI lifetime version at baseline (T1) and the CIDI interval version at follow-up (T2, T3, T5, T6, T7). The CIDI-V is a modified version of the World Health Organization CIDI (WHO-CIDI) (38) that allows for a fully standardized assessment of DSM-IV-TR mental disorders in women (with specifying ICD-10 codes). Psychometric properties of the CIDI were modest to very good (39, 40). Diagnostic interviews were conducted by psychologists having received 1 week of intensive training and conducting a series of supervised interviews. Interviewers were closely monitored throughout the field period by experienced supervisors (clinical psychologists) (36).

Lifetime PTSD was assessed at Baseline (T1) and in Follow-up (T2, T3, T5, T6, and T7) interviews using the questions of the CIDI-V (Section N). Overall, *n* = 25 participants suffered from PTSD prior to or during pregnancy (35).

Birth-related traumatization was assessed at T5, T6, and T7 using adapted questions and lists of section N relating to the birth. It was defined as traumatic birth experience (A1: delivery as traumatic event or A2: intense fear, helplessness, or horror) AND hyperarousal or re-experiencing delivery or avoidance at T5, T6, or T7. Birth-related traumatization was indicated by *N* = 35 women. Of those women, *N* = 19 additionally indicated postpartum depression.

Since only *N* = 4 women indicated both PTSD prior to or during pregnancy and birth-related traumatization, this small group could not be analyzed in more detail.

#### Maternal and Infant Outcomes

*Fear of childbirth during pregnancy and experience of childbirth* was measured based on the German version of the Wijma Delivery Expectancy/ Experience Questionnaire (W-DEQ, Version A, Version B) (41–43). The W-DEQ is a reliable and valid 33-item questionnaire, with scores ranging from ‘not at all' (0) to ‘extremely' (5), yielding a minimum score of 0 and a maximum score of 165 (some items have to be reversed and a higher score indicates more intense fear of childbirth) (44, 45).

*Maternal pregnancy- and child related fears* were assessed at the end of the CIDI-V section on anxiety disorders with embedded questions and lists (37). During the assessments in pregnancy (T1–T3) the participants were instructed to read a list with pregnancy-related fears (e.g., rumination about current pregnancy, fear of labor pain, fear of vaginal delivery) and at T6 and T7 a list with child-related fears was provided (e.g., fear of mistakes concerning child care, fear of financial problems, fear of age-inappropriate development). The participants were asked “Have you ever (“Since the last interview, have you…”) had a strong fear or avoidance of any of the situations/things in the list?” If the participants indicated one or more of these fears, they were asked whether the fear was excessive or stronger compared to other women and to evaluate the burden of the reported fears. Further questions on the presentation of associated anxiety symptoms (e.g., racing heart, sweating, trembling/shaking, dry mouth, difficult breathing, sensation of choking), frequency (sometimes or most of the time vs. only once), and interference with daily live (not at all or somewhat vs. a lot or very much) were asked.

Information on *gestational age, preterm delivery* (<37 + 0 week of gestation), *infant birth weight*, and *mode of delivery* was collected via medical records (46).

*Sexual problems* were assessed at T7 using the German version of the Massachusetts General Hospital Sexual Function Questionnaire (MGH) (47, 48). The MGH is a five-item screening instrument that assesses sexual problems with respect to sexual interest, arousal, orgasm, lubrication, and overall sexual satisfaction: (i) “How has your interest in sex been over the past month?”; (ii) “How has your ability to become sexually aroused or excited been over the past month?”; (iii) “How has your ability to achieve orgasm been over the past month?”; (iv) “How has your ability to become or remain lubricated been over the past month?”; (v) “How would you rate your overall sexual satisfaction over the past month?”. Items are labeled: 1: greater than normal; 2: normal; 3: minimally diminished; 4: moderately diminished; 5: markedly diminished; and 6: totally absent. Based on previous studies (47), a cutoff score of three was used to differentiate normal sexual functioning (≤ 3) from putative sexual problems or dysfunctions (>3) on each item. Concurrent and predictive validity (with respect to sexual functioning and sexual dysfunctions assessed by other self-reports and diagnostic instruments) of the MGH have been shown to be high (47, 49).

*Duration of breastfeeding* and *infant regulatory disorders* were assessed with a structured diagnostic interview (Baby-DIPS) at T5, T6, and T7 (50). *Excessive infant crying* was defined according to the “rule of three” by Wessel et al. (51) as crying for ≥3 h per day on ≥3 days per week for ≥3 weeks (51). *Feeding problems* were defined as any feeding problem (from a list of 16 feeding problems), failure-to-thrive, or mothers worrying (a lot or very much) about infant growth over a period of at least 4 weeks. *Sleeping problems* were defined as difficulties in initiating or maintaining sleep for ≥3 nights per week for ≥3 months while the mother was somewhat, a lot, or very much impaired by her infant's sleeping difficulties between 6 and 16 months (52). Cases that were attributable to a concurrent medical condition were excluded. The Baby-DIPS comprises good to excellent inter-rater reliability as well as high acceptance rates for interviewers and participants (53). Rates and co-occurrence of regulatory problems in the MARI sample were comparable to rates reported by others (52, 54).

*Maternal bonding* was assessed with the German Version of the Postpartum Bonding Instrument (PBQ) at T5 (55). This self-report questionnaire consists of 25 items assessing impaired bonding, rejection and anger, anxiety about care, and risk of abuse (42, 56). The very good psychometric properties were confirmed for the German Version (55). For this analysis, the scales impaired bonding as well as rejection and anger were relevant.

Observations of the mothers and their infants were conducted using standardized observation paradigms to assess infants' temperament (57), neuropsychological development (58), and the quality of infant attachment (59). Standardized observations were conducted by two female psychologists who were blinded to the diagnosis of the mother and had received 2 weeks of training. All observations were recorded by three cameras and supervised to ensure a high assessment quality. Video coding was conducted with the software “Interact” (Mangold).

*Neuropsychological development* was assessed at T6 and T7 with the standardized procedure by Petermann and Renziehausen (58). The tasks allow for an assessment of developmental deficits in different areas (movement control, fine motor skills, visual perception, exploration behavior, receptive and expressive language, cognitive performance). Coding was conducted by graduate and postgraduate psychology students according to the manual guidelines (58).

*Temperament* was measured with the procedures developed by Kagan et al. (60). For the assessment of *infant's reactivity* at T6, the infants sat in a reclining cushioned seat and heard some taped sentences, saw three different colorful mobiles move back and forth, had a cotton swab dipped in dilute butyl alcohol placed under the nostrils, heard a female voice speaking different syllables, and saw a colored umbrella spread out. During these procedures the mother was out of view of the infant. Videotapes were coded for high motor activity (multiple arm, leg, and back movements) and crying (high percentage of time spent crying) in response to the stimuli. Infants were classified as “highly reactive” (high motor activity and crying), “low reactive” (low motor activity and crying), or neither (either high motor activity and low crying amount or the opposite). The assessment of *behavioral inhibition* at T7 involved the presentation of inanimate stimuli to the child (a spinning bingo wheel with noisy objects inside, rotating toys, a puppet show, and sweet and sour tastes). Moreover, strangers (a woman dressed in a white laboratory coat and surgical face mask and a woman with a black cloth over her head and shoulders) attempted to interact with the child. Video coding was conducted by graduate and postgraduate psychology students who were blinded and had received a training of 2 weeks for coding the infant's reactivity and behavioral inhibition (60). The average coding time was 45 min for reactivity and behavioral inhibition. At least 10 videos were coded during training sessions to yield an adequate observer agreement (kappa >0.8) and about 10% of all videos were randomly selected and re-analyzed for quality check to ensure observer agreement with satisfying results (61).

*Attachment:* At T7 mother-infant-dyads participated in the Strange Situation Procedure (59), which consisted of eight episodes, including two brief separations and reunions of the infant from/with the mother. Following the procedures described by Ainsworth et al. (59) and Main and Solomon (62), the attachment group classification was based primarily on the infant's reactions to the mother's return (59, 62). Infants who actively greeted and/or sought contact with the mother upon reunion and returned to exploration within 3 min were classified as secure (Typ B: secure). Infants who actively averted gaze or avoided or ignored the mother immediately upon reunion (Typ A: avoidant) and infants who sought to reunite with the mother but displayed ineffective proximity and contact-seeking behavior, showing anger and active resistance to contact or prolonged fussiness and persistent low-level distress (Typ C: ambivalent/resistant) were classified as insecure. Video coding was conducted by graduate and postgraduate psychology students who were blinded and had received the coding training. The average coding time for attachment videos was 60 min. Cohen's kappa coefficient for the secure and insecure (avoidant and resistant) attachment classifications was based on 20% of randomly selected and independently scored videotapes of the Strange Situation Test (63). Interrater reliability was conducted on 20% of the sample (κ = 1.00 for ABC classification). Final scores for difficult tapes and coder disagreements were based on consensus [for more information see (64)].

### Statistical Analyses

All analyses were performed using STATA version 14 (65). For research question (1) linear and logistic regression analyses were conducted to examine concurrent and prospective associations (Beta, ß and odds ratios, OR) of maternal PTSD prior to or during pregnancy with and without interpersonal trauma with maternal and infant health outcomes (reference: women with neither anxiety nor depressive disorder until T3). For research question (2) linear and logistic regression analyses were conducted to examine betas/ORs including 95% confidence intervals (CI) of maternal birth-related traumatization (with and without depression) with maternal and infant health outcomes (reference: neither anxiety nor depressive disorder prior to or during pregnancy and no birth-related traumatization). Separate analyses were conducted for each outcome and no adjustment for multiple testing was applied, because the individual tests were related to individual hypotheses. Statistical significance was evaluated two-sided at the 5% level (*p* < 0.05).

## Results

To investigate research question 1, analyses were based on the following three diagnostic groups:

- Reference: neither anxiety nor depressive disorder prior to or during pregnancy (until T3) (*N* = 100)
- PTSD prior to or during pregnancy (cumulative lifetime diagnosis until T3) (*N* = 25)
- PTSD prior to or during pregnancy with interpersonal traumata (cumulative lifetime diagnosis until T3) (*N* = 17)

Women with PTSD prior to or during pregnancy were characterized by a lower educational level (education: ≤ 10 years: reference: 25.0%, PTSD prior to or during pregnancy: 52.0%; Chi^2^=6.891, *p* = 0.009) compared to women with neither anxiety nor depressive disorder until delivery (Table 1). Moreover, women with PTSD prior to or during pregnancy reported more often nicotine consumption during pregnancy (16.0% vs. 3.0%; Chi^2^=6.394, *p* = 0.001) and lower social support during pregnancy (F-Sozu: M=4.1, SD=0.7 vs. M=4.5, SD=0.4; *t* = 3.122*, p* > 0.001) as compared to the reference group. There were no significant differences between the groups with regard to age, marital status, and monthly household income (Table 1).

**Table 1:** Sociodemographic and gynecological characteristics of women with any PTSD until T3 (with/without interpersonal trauma) (*N* = 25) as compared to women with neither anxiety nor depressive disorder until T3 (*N* = 100).

	**Women with neither anxiety nor depressive disorder until T3** **(*N* = 100)**	**PTSD prior to or during pregnancy with/without interpersonal trauma (*N* = 25)**	**Significant group differences**
	***N*, % or M, SD**	***N*, % or M, SD**	
Age (M, SD)	27.9 (4.6)	27.1 (3.7)	
Educational status (*n*, %)			
Lower education (≤ 10 years)	25 (25.0)	**13 (52.0)**	**Chi**^2^**=6.891**, ***p*** **=** **0.009**
Higher education (>10 years)	75 (75.0)	**12 (48.0)**	
Marital status (*n*, %)			
Not married	64 (64.0)	18 (72.0)	
Married	36 (36.0)	7 (28.0)	
Cohabitation (*n*, %)			
Not living together	6 (6.0)	0 (0.0)	
Living together	94 (94.0)	25 (100.0)	
Working time at baseline (*n*, %)			
Full-time job	37 (37.0)	8 (32.0)	
Part-time job	26 (26.0)	4 (16.0)	
Currently not working	37 (37.0)	13 (52.0)	
Monthly household income after taxes (*n*, %)	
<500€	9 (9.0)	1 (4.0)	
500–1,500€	34 (34.0)	11 (44.0)	
1,500–2,500€	27 (27.0)	9 (36.0)	
2,500–3,500€	19 (19.0)	2 (8.0)	
3,500–4,500€	8 (8.0)	2 (8.0)	
>4,500€	3 (3.0)	0 (0.0)	
Parity (*n*, %)	
Primipara	60 (60.0)	11 (44.0)	
Multipara	40 (40.0)	14 (56.0)	
Pregnancy planned/ desired: yes (*n*, %)	86 (94.5)	22 (95.7)	
Prior spontaneous abortions (*n*, %)			
No	79 (79.0)	16 (64.0)	
Yes	21 (21.0)	9 (36.0)	
Any nicotine consumption during pregnancy (*n*, %)	3 (3.0)	**4 (16.0)**	**Chi**^2^**=6.394**, ***p*** **=** **0.011**
Any alcohol consumption during pregnancy (*n*, %)	31 (31.0)	7 (28.0)	
Social support during pregnancy (F-SozU, T2) (M, SD)	4.5 (0.4)	**4.1 (0.7)**	***t*** **=** **3.122**, ***p*** **>** **0.001**
Partnership quality during pregnancy (PFB, T2) (M, SD)	72.8 (10.5)	68.5 (18.1)	

*T3, MARI assessment during third trimester of pregnancy; REF, reference; PTSD, post-traumatic stress disorder; n, number; %, percentage; M, mean; SD, standard deviation; Chi^2^, Chi-squared test; t, t-test; p, p-value. Bold: significant group differences*.

Table 2 shows maternal and infant outcomes in women with PTSD prior to or during pregnancy compared with or without interpersonal trauma (*N* = 25) to women with neither anxiety nor depressive disorder until T3 (*N* = 100). Women with history of PTSD reported more fear of childbirth and fear of epidural anesthesia and perceived delivery more often as traumatic (including re-experiencing delivery later on). Moreover, infants of women with PTSD prior to delivery and interpersonal trauma (*N* = 17) were at higher risk for feeding problems.

**Table 2:** Maternal and infant outcomes in women with PTSD prior to or during pregnancy (with interpersonal trauma) compared to women with neither anxiety nor depressive disorder until T3.

	**Reference group: Neither anxiety nor depressive disorder prior to or during pregnancy (until T3) (*N* = 100)**	**PTSD prior to or during pregnancy (w/wo interpersonal trauma) (*****N*** **=** **25)**		**PTSD prior to or during pregnancy with interpersonal traumata (until T3) (*****N*** **=** **17)**	
	***N*, % or M, SD**	***N*, % or M, SD**	**OR (95%CI) or ß (95%CI)**	***N*, % or M, SD**	**OR (95%CI) or ß (95%CI)**
**Maternal health during pregnancy and birth experience**					
Fear of childbirth (W-DEQ-A, T3) (M, SD)	57.9 (21.5)	**69.3 (22.1)**	**ß** **=** **11.40 (1.21–21.59)**	**69.7 (19.0)**	**ß** **=** **11.80 (0.08–23.51)**
Birth experience (W-DEQ-B, T4) (M, SD)	62.8 (21.8)	67.3 (18.2)		65.8 (18.8)	
Pregnancy-related anxiety (*n*, %)					
Fear of labor pain	19 (19.0)	8 (32.0)		5 (29.4)	
Fear of vaginal delivery	26 (26.0)	10 (40.0)		8 (47.1)	
Fear of perineal rupture/episiotomy	27 (27.0)	6 (24.0)		5 (29.4)	
Fear of epidural anesthesia	8 (8.0)	**10 (40.0)**	**OR** **=** **7.67 (2.61–22.53)**	**7 (41.2)**	**OR** **=** **8.05 (2.41–26.89)**
Excessive pregnancy-related fear	7 (7.0)	*5 (20.0)*		3 (17.7)	
Presentation of anxiety symptoms	8 (8.0)	4 (16.0)		3 (17.7)	
Interference with daily live	3 (3.0)	**6 (24.0)**	**OR** **=** **10.21 (2.35–44.43)**	**3 (17.7)**	**OR** **=** **6.93 (1.27–37.76)**
Evaluation of fears as frequent	9 (9.0)	4 (16.0)		3 (17.7)	
Mode of delivery (*n*, %)					
Spontaneous delivery	78 (85.7)	20 (87.0)		14 (87.5)	
C-section/ Operative vaginal delivery	13 (14.3)	3 (13.4)		2 (12.5)	
Support and control during delivery (M, SD)					
Internal control (SCIB)	32.2 (8.1)	28.7 (9.2)		27.9 (9.4)	
External control (SCIB)	38.1 (8.9)	38.0 (8.2)		38.4 (8.7)	
Support (SCIB)	46.9 (8.6)	46.7 (11.3)		44.6 (12.0)	
DSM-VI criteria for traumatic birth experience (*n*, %)					
A1: delivery as traumatic event	11 (12.1)	**9 (42.9)**	**OR** **=** **5.45 (1.87–15.90)**	**6 (42.9)**	**OR** **=** **5.45 (1.59–18.70)**
A2: intense fear, helplessness, or horror	15 (16.5)	**8 (38.1)**	**OR** **=** **3.12 (1.10–8.82)**	**6 (42.9)**	**OR** **=** **3.80 (1.15–12.55)**
B: re–experiencing delivery	1 (1.0)	**4 (16.0)**	**OR** **=** **18.85 (2.00–177.37)**	**2 (11.8)**	**OR** **=** **13.20 (1.13–154.68)**
C: avoidance of recollection due to delivery	0 (0.0)	0 (0.0)	omitted	0 (0.0)	omitted
E: alterations in arousal following delivery	6 (6.0)	2 (8.0)		0 (0.0)	omitted
Any postpartum anxiety disorder (*n*, %)	8 (8.8)	**11 (47.8)**	**OR** **=** **9.51 (3.19–28.39)**	**7 (43.8)**	**OR** **=** **8.07 (2.37–27.49)**
Postpartum depression (*n*, %)	0 (0.0)	7 (30.4)	omitted	5 (31.3)	omitted
**Infant health outcomes**					
Gestational age (M, SD)	39.5 (1.5)	39.4 (1.2)		39.4 (1.2)	
Birth weight (M, SD)	3,403.2 (58.1)	3.510.7 (440.7)		3,494.1 (440.6)	
Excessive crying (*n*, %)	6/91 (6.6)	3/23 (13.0)		2/16 (12.5)	
Feeding problems (*n*, %)	24/91 (26.4)	10/23 (43.5)		**9/16 (56.3)**	**OR** **=** **3.59 (1.20–10.70)**
Sleeping problems (*n*, %)	9/91 (9.9)	4/23 (17.4)		4/16 (25.0)	
Neuropsychological development (T6) (M, SD)	11.6 (0.8)	11.6 (0.6)		11.6 (0.6)	
Neuropsychological development (T7) (M, SD)	13.3 (1.8)	13.6 (1.6)		13.1 (1.7)	
Temperament: highly reactive (T6) (*n*, %)	9/83 (10.8)	5/22 (22.7)		4/15 (26.7)	
Temperament: Behavioral Inhibition (T7) (*n*, %)	38/84 (45.2)	5/20 (25.0)		5/13 (38.5)	
Insecure attachment (Strange Situation) (T7) (*n*, %)	29/82 (35.4)	7/20 (35.0)		4/13 (30.8)	

*T3, 3rd assessment during third trimester of pregnancy; T5, 5th assessment 2 months postpartum; T6, 6th assessment 4 months postpartum; T7, 7th assessment 16 months postpartum; REF, reference; Any PTSD until T3, women with any PTSD until T3, Any PTSD + man-made trauma until T3, women with any PTSD with manmade trauma until T3, W-DEQ, Wijma Delivery Expectancy/Experience Questionnaire; PBQ, Postpartum Bonding Questionnaire; N, number; %, percentage; M, mean; SD, standard deviation; OR, odds ratio; 95%CI, 95% confidence interval. Bold: significant group differences*.

To investigate research question 2, analyses were based on the following three diagnostic groups:

- Reference: neither anxiety nor depressive disorder prior to or during pregnancy (until T3) and no birth-related traumatization (traumatic birth experience (A1 or A2) AND no hyperarousal/ re-experiencing/ avoidance) (*N* = 70)
- birth-related traumatization (traumatic birth experience (A1 or A2) AND hyperarousal or re-experiencing or avoidance) (*N* = 35)
- birth-related traumatization (traumatic birth experience (A1 or A2) AND hyperarousal or re-experiencing or avoidance) AND depression (*N* = 19)

Table 3 shows maternal and infant outcomes in women with birth-related traumatization compared to women with neither anxiety nor depressive disorder until T3 and no birth-related traumatization. A higher risk for child-related fears, postpartum anxiety disorders, and sexual problems was reported by women with birth-related traumatization. Moreover, infants of mothers with birth-related traumatization and postpartum depression were at higher risk for feeding and sleeping problems.

**Table 3:** Maternal and infant outcomes in women with birth-related traumatization (and depression) after delivery compared to women with neither anxiety nor depressive disorder until T3 and no birth-related traumatization.

	**Reference group: Neither anxiety nor depressive disorder until T3 and no birth-related traumatization (*N* = 70)**	**Birth-related traumatization: Traumatic birth experience (A1/ A2)** **+** **(hyperarousal/ re-experiencing/ avoidance) (*****N*** **=** **35)**		**Birth-related traumatization: Traumatic birth experience (A1/ A2)** **+** **(hyperarousal/ re-experiencing/ avoidance)** **+** **depression (*****N*** **=** **19)**	
	***N*, % or M, SD**	***N*, % or M, SD**	**OR (95%CI) or ß (95%CI)**	***N*, % or M, SD**	**OR (95%CI) or ß (95%CI)**
**Maternal health during postpartum period**					
Duration of breastfeeding (M, SD)	9.4 (4.6)	9.6 (4.5)		8.8 (4.7)	
Maternal impaired bonding (T5) (PBQ) (M,SD)	4.3 (4.1)	5.3 (4.3)		5.5 (4.1)	
Maternal rejection and anger (T5) (PBQ) (M,SD)	1.5 (2.0)	1.7 (2.2)		1.8 (1.9)	
Child-related fears/ anxiety (*n*, %)					
Fear of mistakes concerning child care	9 (12.9)	**11 (31.4)**	**OR** **=** **3.11 (1.14–8.44)**	6 (31.6)	
Fear of mistakes concerning child feeding	12 (17.1)	**14 (40.0)**	**OR** **=** **3.22 (1.29–8.07)**	7 (36.8)	
Fear of financial problems	2 (2.9)	2 (5.7)		2 (10.5)	
Fear of mistakes concerning child rearing	3 (4.3)	**10 (28.6)**	**OR** **=** **8.93 (2.27–35.14)**	**6 (31.6)**	**OR** **=** **10.31 (2.28–46.56)**
Fear concerning quality of day care*	7 (10.3)	8 (23.5)		3 (16.7)	
Fear that child suffers from familial conflicts*	0 (0.0)	4 (11.8)	omitted	1 (5.6)	omitted
Fear of separation from child	3 (4.4)	**7 (20.6)**	**OR** **=** **5.62 (1.35–23.36)**	3 (16.7)	
Fear of age-inappropriate infant development	5 (7.1)	5 (14.3)		**5 (26.3)**	**OR** **=** **4.64 (1.18–18.23)**
Fear of viral or other infection/ disease	8 (11.4)	8 (22.9)		4 (21.1)	
Fear of infant injury	11 (15.7)	9 (25.7)		3 (15.8)	
Fear of infant death	13 (18.6)	**16 (45.7)**	**OR** **=** **3.69 (1.51–9.06)**	**9 (47.4)**	**OR** **=** **3.95 (1.34–11.66)**
Evaluation of fear as excessive	30 (42.9)	**23 (65.7)**	**OR** **=** **2.56 (1.10–5.94)**	13 (68.4)	
Presentation of anxiety symptoms	7 (10.0)	**10 (28.6)**	**OR** **=** **3.60 (1.23–10.51)**	5 (26.3)	
Evaluation of fears as frequent	6 (8.6)	**9 (25.7)**	**OR** **=** **3.69 (1.19–11.42)**	4 (21.1)	
Interference with daily life	2 (2.86)	**5 (14.3)**	**OR** **=** **5.67 (1.04–30.87)**	**4 (21.1)**	**OR** **=** **9.07 (1.52–54.15)**
Any anxiety disorder postpartum	6 (8.6)	**14 (40.0)**	**OR** **=** **7.11 (2.43–20.86)**	**8 (42.1)**	**OR** **=** **7.76 (2.25–26.72)**
Postpartum depression	0 (0.0)	19 (54.3)	omitted	*19 (100.0)*	*group definition*
Sexual problems (T7) (M, SD)					
Sexual interest	3.2 (1.4)	**3.9 (1.3)**	**ß** **=** **0.76 (0.18–1.33)**	**4.2 (1.2)**	**ß** **=** **1.02 (0.30–1.75)**
Sexual arousal	2.8 (1.4)	**3.4 (1.4)**	**ß** **=** **0.60 (0.02–1.19)**	3.5 (1.4)	
Orgasm	2.7 (1.4)	**3.6 (1.7)**	**ß** **=** **0.90 (0.26–1.53)**	**3.8 (1.8)**	**ß** **=** **1.10 (0.29–1.91)**
Lubrication	2.3 (0.9)	**3.0 (1.4)**	**ß** **=** **0.76 (0.28–1.24)**	**2.9 (1.4)**	**ß** **=** **0.67 (0.09–1.25)**
Overall sexual satisfaction	2.8 (1.3)	**3.9 (1.5)**	**ß** **=** **1.1 (0.50–1.67)**	**4.4 (1.5)**	**ß** **=** **1.57 (0.84–2.29)**
**Infant Health and infant outcomes**					
Excessive crying (*n*, %)	5/70 (7.1)	4/35 (11.4)		3/19 (15.8)	
Feeding problems (*n*, %)	17/70 (24.3)	15/35 (42.9)		**12/19 (63.2)**	**OR** **=** **5.34 (1.81–15.74)**
Sleeping problems (*n*, %)	8/70 (11.4)	9/35 (25.7)		**8/19 (42.1)**	**OR** **=** **5.64 (1.75–18.18)**
Neuropsychological development (T6) (M, SD)	11.4 (0.9)	11.6 (0.8)		11.7 (0.8)	
Neuropsychological development (T7) (M, SD)	13.3 (1.9)	13.1 (2.0)		12.9 (2.3)	
Temperament: highly reactive (T6) (*n*, %)	7/63 (11.1)	5/32 (15.6)		2/17 (11.8)	
Temperament: behavioral Inhibition (T7) (*n*, %)	32/66 (48.5)	14/31 (45.2)		7/16 (43.8)	
Insecure attachment (Strange Situation) (T7) (*n*, %)	26/65 (40.0)	15/31 (48.4)		8/16 (50.0)	

T5, 5th assessment 2 months postpartum; T6, 6th assessment 4 months postpartum; T7, 7th assessment 16 months postpartum; REF, reference; A1, DSM–IV PTSD criteria A1; A2, DSM–IV PTSD criteria A2; PBQ, Postpartum Bonding Questionnaire; N, number; %, percentage; M, mean; SD, standard deviation; OR, odds ratio; ß, Beta-coefficient; 95%CI, 95% confidence interval; ^*^Data of N = 267 participants available only. Bold: significant group differences.

## Discussion

This prospective longitudinal study demonstrated that (1) women with PTSD prior to or during pregnancy presented with a disadvantageous psycho-social situation, reported more pronounced fear of childbirth, and indicated more often a traumatic birth experience compared to women without anxiety and depressive disorder prior to delivery. Moreover, infants of women with PTSD and interpersonal trauma, had a higher risk for feeding problems. (2) Women with birth-related traumatization indicated numerous adverse outcomes (e.g., postpartum anxiety and depression, child-related fears, sexual problems), and in case of additional postpartum depression, the infants were more often affected by feeding and sleeping problems.

The first research question examined the associations of PTSD prior to or during pregnancy with specific maternal and infant outcomes. As expected and in line with previous research, women with PTSD prior to or during pregnancy reported more often pregnancy- and child-related fears and they were further at higher risk for a traumatic birth experience compared to women without anxiety and depressive disorder prior to delivery (12). Moreover, those women presented with a disadvantageous psycho-social situation (lower educational level, less social support) and indicated more often nicotine consumption during pregnancy (66, 67). These factors can be associated with a cascade of behavioral health and neuroendocrine changes that may not only have negative consequences for the affected women, but also for the infants (68, 69). Regarding the current discussion on intergenerational transmission of trauma effects it is particularly interesting that women with lifetime PTSD reported more pronounced pregnancy-related fears (70). Yehuda and Lehrner recently highlighted that preconception trauma in parents might be associated with epigenetic changes and developmentally programmed effects that can result from offspring's early environmental in utero and postnatal exposures leading to an increased susceptibility of the infant to later environmental distress (70, 71). We found a higher risk for infant regulatory disorders (feeding problems) in mothers with PTSD prior to or during pregnancy and interpersonal traumata (2, 28, 29, 31, 32). However, no significant associations were seen for e.g., temperament, neuropsychological development, or bonding. One explanation could be that lifetime PTSD was not present anymore during the perinatal period in some of the women due to recovery or successful therapy. However, it still seems to be relevant for the development of infant feeding problems in case of interpersonal trauma. Another explanation might be the limited statistical power to detect differences between the respective groups (see limitations section).

The second research question pertains to birth-related traumatization. As expected, women with birth-related traumatization reported more often sexual problems as compared to the reference group (12). Given that sexual problems impede the partnership or interfere with further family planning, affected women might profit from targeted early interventions supporting them and their partners to successfully cope with sexual dysfunctions and associated problems (18, 72). In line with previous evidence, women with birth-related traumatization reported more often postpartum anxiety and depressive disorders as compared to the reference group (12). This is important to note, since traumatic birth experience might change the way affected mothers interact with their infants, especially if postpartum depression is also present (e.g., avoiding contact with or being emotionally unavailable for the infant) (73, 74). Moreover, we saw a higher risk for infant regulatory disorders (sleeping and feeding problems) in mothers with birth-related traumatization and postpartum depression. This was in line with the results by Garthus-Niegel and colleagues who reported an association of PTSD and impaired child sleep (75). However, no significant associations were seen for e.g., temperament, neuropsychological development, or bonding. This was surprising since other prospective studies found maternal postpartum PTSD to be associated with poorer cognitive and social-emotional development in infants up to 2 years (19, 30). However, Garthus-Niegel et al. (19) did not find significant associations with other domains of child development (fine/gross motor or communication development) which was also not seen in our sample (19).

Taken together, our findings highlight the importance of early targeted prevention and intervention for affected women (20) and the peripartum period represents an opportunity to interrupt the pattern of intergenerational transmission of trauma. Knowledge of PTSD prior to or during pregnancy that is also associated with a higher risk of traumatic birth experience (e.g., experience of fear, helplessness or horror, or re-experiencing delivery) is crucial for health-care providers to be alert of when they treat these high-risk women (20, 76). Moreover, trauma-informed interventions should be developed and tested especially for women with PTSD and a history of interpersonal trauma or comorbid depression (74, 77). Targets that should be addressed are prevention of re-traumatization during delivery, improvement of maternal partnership problems and social support, and sensitivity training to encourage mother-child-interaction and bonding/ attachment (78–80). First evidence shows that cognitive behavioral therapy (81) and Eye Movement Desensitization and Reprocessing approaches (82–84) may improve PTSD status but require investigation in randomized controlled trials (85). Debriefing may only be successful if women are requesting it themselves (86). Since PTSD can also affect the developing relationship with the child, mother-infant bonding problems should also be addressed (20, 23). For some mothers, their infants are a reminder of their traumatic birth and may therefore trigger avoidance behaviors (i.e., non-initiation of breastfeeding) (21). Here, Mother Baby Connections, a program involving interaction therapy, has brought first promising results (87).

### Strengths and Limitations

A particular feature of this investigation was the recruitment already during early pregnancy and the consideration of lifetime diagnostic status. To our knowledge this is the first study investigating both, lifetime PTSD and birth-related traumatization, with associated maternal and infant outcomes. Strengths of this study include the prospective multi-wave design and the long follow-up period. Findings are limited by small cell sizes in some clinical groups. This limits power to detect differences between the respective groups. Thus, the absence of significant differences between infants of mothers with no anxiety and depressive disorder as compared to mothers with lifetime PTSD or birth-related traumatization regarding, e.g., temperament, neuropsychological development, or bonding, should not be interpreted as indicative of negative results. It was also not possible to explore the putative cumulative risk of lifetime PTSD and birth-related traumatization on maternal and infant outcomes. Moreover, sample size prohibited examination of third variables that might also be relevant (e.g., income, education, birth outcomes) (88).

## Conclusion

The study shows that PTSD is a highly debilitating disorder and affected (expectant) mothers might profit from early interventions already during pregnancy and the initial postpartum period (89). Our results highlight the crucial role of early identification and treatment of affected women. Women's health care providers should screen for PTSD and subsyndromal posttraumatic stress symptoms in routine assessments during and after pregnancy, especially in women with a reported history of trauma. Such screening will allow women to receive needed treatment and referrals and mitigate the potentially negative sequelae of PTSD (14). However, screening without sufficient services is not helpful and could be triggering for both professionals and families. Thus, a fully trauma informed perinatal system and effective trauma specific interventions during pregnancy and immediately after traumatic births is warranted to ensure that the families receive help for parenting and early bonding (77).

Finally, this *post-hoc* analysis was conducted with the aim of generating hypotheses for future research. Larger prospective studies are warranted to examine why interpersonal trauma is especially harmful for perinatal outcomes, and to disentangle whether this is driven by the interpersonal trauma in general, or trauma related to mothers' own childhood attachment relationships. It will be important to examine the role of hormonal alterations and other biological or psychosocial factors in the context of perinatal PTSD and to include a comprehensive assessment on psychotherapeutic and pharmacological treatments. Finally, it is important to note, that paternal PTSD after childbirth is a highly understudied and unrecognized problem that should receive more attention.

## Data Availability Statement

The datasets presented in this article are not readily available because after consulting the Ethics Committee and due to the sensitive nature of the questions asked in this study, participants were assured that all raw data would remain confidential and would not be shared. Therefore, no openly assessable data files are attached. Further information on the data can be obtained from the corresponding author (JM, email: julia.martini@tu-dresden.de), the Ethics Committee of the Medical Faculty of the Technische Universität Dresden (email: ethikkommission@mailbox.tu-dresden.de), and the Institute of Clinical Psychology and Psychotherapy of the Technische Universität Dresden.

## Ethics Statement

The studies involving human participants were reviewed and approved by Ethics Committee of the Technische Universität Dresden (No: EK 94042007). Written informed consent to participate in this study was provided by the participants' legal guardian.

## Author Contributions

JM conceptualized and designed the study, designed the data collection instruments, collected data for the study, carried out the statistical analyses and the interpretation of the data, drafted the initial manuscript, and approved the final manuscript as submitted. EA, SK, KW, JR, and SG-N critically reviewed the manuscript and approved the final manuscript as submitted. All authors contributed to the article and approved the submitted version.

## Funding

The MARI study was funded by the Institute of Clinical Psychology and Psychotherapy, Technische Universität Dresden, Germany and supported in part by the Lundbeck Institute Skodsborg, Denmark. Parts of the field work were additionally supported by the Friends and Sponsors (Gesellschaft der Freunde und Förderer) of the Technische Universität Dresden, Germany.

## Conflict of Interest

The authors declare that the research was conducted in the absence of any commercial or financial relationships that could be construed as a potential conflict of interest.

## Publisher's Note

All claims expressed in this article are solely those of the authors and do not necessarily represent those of their affiliated organizations, or those of the publisher, the editors and the reviewers. Any product that may be evaluated in this article, or claim that may be made by its manufacturer, is not guaranteed or endorsed by the publisher.
